# Increased *Toxoplasma gondii* Intracellular Proliferation in Human Extravillous Trophoblast Cells (HTR8/SVneo Line) Is Sequentially Triggered by MIF, ERK1/2, and COX-2

**DOI:** 10.3389/fmicb.2019.00852

**Published:** 2019-04-24

**Authors:** Iliana Claudia Balga Milian, Rafaela José Silva, Camilla Manzan-Martins, Bellisa Freitas Barbosa, Pamela Mendonça Guirelli, Mayara Ribeiro, Angelica de Oliveira Gomes, Francesca Ietta, José Roberto Mineo, Priscila Silva Franco, Eloisa Amália Vieira Ferro

**Affiliations:** ^1^ Laboratory of Immunophysiology of Reproduction, Institute of Biomedical Science, Federal University of Uberlândia, Uberlândia, Brazil; ^2^ Department of Morphology, Federal University of the Triângulo Mineiro, Uberaba, Brazil; ^3^ Department of Life Sciences, University of Siena, Siena, Italy; ^4^ Laboratory of Immunoparasitology, Institute of Biomedical Sciences, Federal University of Uberlândia, Uberlândia, Brazil

**Keywords:** *Toxoplasma gondii*, macrophage migration inhibitor factor (MIF), CD44, ERK1/2 phosphorylation, COX-2

## Abstract

Macrophage migration inhibitory factor (MIF) is a potent pro-inflammatory cytokine, which mediates the regulation of diverse cellular functions. It is produced by extravillous trophoblastic cells and has been found to be involved in the pathogenesis of diseases caused by some protozoa, including *Toxoplasma gondii*. Previous studies demonstrated the ability of *T. gondii* to take advantage of MIF action in human trophoblast cells. However, MIF action in *T. gondii*-infected extravillous trophoblastic cells (HTR8/SVneo cell line) has not been fully investigated. The present study aimed to investigate the role of MIF in *T. gondii*-infected HTR8/SVneo cells and verify the intracellular signaling pathways triggered by this cytokine. We found that *T. gondii* increased MIF production by HTR8/SVneo cells, and by contrast, MIF inhibition, by ISO-1, led to a significant decrease in *T. gondii* proliferation and CD74 expression in HTR8/SVneo cells. Moreover, in infected HTR8/SVneo cells, the addition of recombinant MIF (rMIF) increased CD44 co-receptor expression, ERK1/2 phosphorylation, COX-2 expression, and IL-8 production, which favored *T. gondii* proliferation. Our findings indicate that *T. gondii* can use MIF to modulate important factors in HTR8/SVneo cells, being a possible explanation for the higher susceptibility of extravillous trophoblast cells than other trophoblast cell populations.

## Introduction

*Toxoplasma gondii,* the agent of toxoplasmosis, is an obligate intracellular protozoan parasite and a member of the phylum Apicomplexa (Schluter et al., (2014). The parasite infects many types of vertebrates, including humans, and it is highly prevalent throughout the world (Tenter et al., 2000; Melo et al., 2011). Infection in humans is frequently asymptomatic, but it can lead to severe disease in immunocompromised patients and congenitally infected children, leading to several manifestations, such as retinochoroiditis and miscarriage during the first trimester of pregnancy (Tenter et al., 2000; Unno et al., 2010; Vasconcelos-Santos, 2012).

Successful gestation is associated with no rejection of paternal antigens from the mother, with predominant secretion of anti-inflammatory mediators (Vargas-Villavicencio et al., 2009). The Th2 cytokine profile is favorable for fetal tolerance but at the same time becomes favorable to *T. gondii* replication (Vargas-Villavicencio et al., 2009), increasing the rate of vertical transmission of the parasite (Remington et al., 2010). Therefore, in the maternal-fetal interface, a complex paradigm is established between preserving the pregnancy or triggering a potent inflammatory response to control the parasite. The classical immune response to *T. gondii* is based on a pro-inflammatory profile, with the production of pro-inflammatory cytokines, such as interleukin (IL)-12, which is produced by macrophages and dendritic cells (DCs) in response to Toll-like receptors (TLRs; Yarovinsky, 2014), in addition to interferon gamma (IFN-γ) released by T cells (Murakami et al., 2002). Another cytokine with a key role in *T. gondii* infection is macrophage migration inhibitory factor (MIF), produced by different cell types and tissues (Bernhagen et al., 1998). MIF is a pro-inflammatory cytokine, and it was identified by Bloom and Bennett (1966) and David (1966). MIF has been shown to participate in both innate and adaptive immune responses (Bloom and Bennett, 1966; David, 1966; Calandra et al., 1995; Calandra and Roger, 2003; Larson and Horak, 2006; Kudrin and Ray, 2008).

Previous studies have observed the involvement of MIF in the maternal-fetal environment during *T. gondii* infection. A study using MIF^−/−^ mice demonstrated that these animals were susceptible to *T. gondii* infection (Flores et al., 2008), and the absence of MIF may trigger local and systemic inflammation, tissue damage, and death (Cavalcanti et al., 2011), demonstrating the significant role that MIF plays in controlling *T. gondii* infection. Other researchers also observed the participation of MIF in some first-trimester explants treated with total *Toxoplasma* antigen (STAg), illustrating that MIF may play an essential role as an autocrine/paracrine mediator in placental infection caused by *T. gondii* (Ferro et al., 2008). Another study evaluated the effect of MIF in human placental explants infected with *T. gondii*, demonstrating that controlling the infection depends on gestational age. In first-trimester villous explants, MIF is upregulated, and it can control *T. gondii* infection, whereas a lack of MIF upregulation, after infection, in third-trimester placental explants may be related to a higher susceptibility to infect at this gestational stage (Gomes et al., 2011).

In extravillous trophoblast cells, elevated levels of MIF, its receptor, CD74, and co-receptor, CD44, are expressed when compared to cytotrophoblast cells (Takahashi et al., 2014). CD44 is one of the key molecules that regulate microenvironment interactions (Al-Hajj et al., 2003). This co-receptor has been recognized as one of the key cell surface markers for many cells. Since CD44 does not have intrinsic kinase activity, intracellular signaling is modulated by interaction with other components of signaling transduction (Ponta et al., 2003). The binding of MIF to the receptor/co-receptor complex (CD74/CD44) activates intracellular signaling leading to the regulation of gene transcription and subsequent expression of effector molecules, such as extracellular regulated kinases 1/2 (ERK 1/2) (Subbannayya et al., 2016). ERK 1/2 phosphorylation triggers cyclooxygenase-2 (COX-2) expression and production of lipid mediators, such as prostaglandins (PGEs; Calandra and Roger, 2003; Wang and Dey, 2005). Initial studies have suggested that MIF, *via* binding to CD74 and the MAPK signaling pathway, significantly upregulates the activation of ERK 1/2, which participates in the activation of cyclooxygenases, especially COX-2 (Wang and Dey, 2005) and IL-8 production (Mahdian et al., 2015). Our previous study demonstrated that MIF triggers ERK 1/2 and prostaglandin E_2_ (PGE_2_) production in human villous trophoblast cells (BeWo cell line) in a dose-dependent manner (Barbosa et al., 2014). In addition, ERK 1/2 and PGE_2_ were able to upregulate *T. gondii* replication in BeWo cells, demonstrating the beneficial effect of low doses of MIF and its intracellular pathway during infection by *Toxoplasma* in human villous trophoblast cells (Barbosa et al., 2014). However, no study has been conducted involving the intracellular mechanisms triggered by MIF in human extravillous trophoblast cells infected with *T. gondii*.

Considering that MIF is important in the immune response against *T. gondii*, systemically and during gestation, and that the role of MIF in infected human extravillous trophoblast cells is unclear, the present study aimed to verify the functional role of MIF in HTR8/SVneo cells. HTR8/SVneo cells, a model of human extravillous trophoblast cells, are widely used to evaluate the migration and proliferation processes of extravillous trophoblast cells (Ko et al., 2013), to understand the factors involved during preeclampsia (Liu et al., 2018), and to examine the cellular effects of MIF in promoting cell migration and invasion (Krivokúca et al., 2014). For this reason, we verified the expression of the MIF receptor, intracellular proliferation of the parasite, and intracellular pathways triggered by MIF in HTR8/SVneo cells, using human recombinant MIF and ISO-1, a potent inhibitor of MIF activity according to the previous studies (Lubetsky et al., 2002; Liang et al., 2010; Sommerville et al., 2013; Krivokúca et al., 2014).

## Materials and Methods

### Cell Culture

The human extravillous trophoblast cell line, HTR8/SVneo, was obtained from villous explants at early pregnancy and was a gift from Dr. Estela Bevilacqua (University of São Paulo, SP, Brazil). These cells were cultured in 25-cm^2^ culture flasks in RPMI 1640 medium (Cultilab, Campinas, SP, Brazil), supplemented with 100 U/ml penicillin, 100 μg/ml streptomycin (Sigma-Aldrich Chemical Co., St. Louis, MO, USA), and 10% heat-inactivated fetal calf serum (FBS) (Cultilab) in a humidified incubator at 37°C and 5% CO_2_ (Castro et al., 2013; Guirelli et al., 2015).

### Parasites

Tachyzoites of the *T. gondii* 2F1 clone, which constitutively expressed cytoplasmic β-galactosidase and are derived from the RH strain, were a gift from Dr. Vern Carruthers (Medical School of Michigan University, USA). These parasites were propagated in human choriocarcinoma cells (BeWo cells) maintained in RPMI 1640, medium supplemented with penicillin (100 U/ml), streptomycin (100 μg/ml), and 2% FBS at 37°C and 5% CO_2_ (Angeloni et al., 2013; Barbosa et al., 2015).

### Cell Viability

Cell viability was evaluated using a tetrazolium salt colorimetric (MTT) assay (Mosmann, 1983) to verify whether human recombinant MIF (rMIF), ISO-1 (an inhibitor of MIF production), or *T. gondii* would be toxic to HTR8/SVneo cells. For this purpose, HTR8/SVneo cells were cultured in 96-well plates (1 × 10^4^ cells/well/200 μl), and after 24 h at 37°C and 5% CO_2_, the cells were treated with rMIF (200 ng/ml, Bioscience, San Diego, CA, USA) or ISO-1 (200 μg/ml, Merck KGaA©, Darmstadt, Germany) or left untreated. After 24 h, a portion of the cells was infected by *T. gondii* in the proportion of three parasites per cell (3:1) for additional 24 h, while the other cells were not infected. As a control, HTR8/SVneo cells were only treated with medium. Since ISO-1 was diluted in DMSO, we treated the cells with 2% DMSO to verify whether DMSO could be toxic to HTR8/SVneo cells. After treatments and/or infection, the supernatants were removed, and the cells were washed with medium and pulsed with 10 μl of tetrazolium salt colorimetric (MTT, Sigma). The plates were incubated under standard culture conditions for 3 h, and the formazan crystals produced by viable cells were solubilized by 10% sodium dodecyl sulfate (SDS) and 50% N, N-dimethylformamide. The optical density was determined at 570 nm using a plate reader (Titertek Multiskan Plus, Flow Laboratories, McLean, VA, USA), and the results were expressed as the percentage of viable cells in relation to control (100% cell viability). Five independent experiments were performed in triplicate for each condition.

### Cytokines Production in Uninfected and *T. gondii*-Infected HTR8/SVneo Cells

First, we verified extracellular MIF production in HTR8/SVneo. For this purpose, HTR8/SVneo cells were cultured in six-well plates (5 × 10^5^ cells/well/1000 μl), and after 24 h at 37°C and 5% CO_2_, a portion of the cells was treated with 200 ng/ml rMIF or 200 μg/ml ISO-1 for an additional 24 h, while the other cells were left untreated. Then, a portion of the cells was infected by *T. gondii* (3:1) and incubated for 24 h, while the other cells were not infected. As a control, HTR8/SVneo cells were only treated with medium. After treatments and/or *T. gondii* infection, the cell-free supernatants were collected for measurement of MIF and IL-8 cytokines by enzyme-linked immunosorbent assay (ELISA). MIF and IL-8 were measured using a sandwich ELISA according to the manufacturer’s instructions (R&D Systems, Minneapolis, MN, USA; BD Bioscience). The data were shown in pg/ml, and the limit of detection was according to the last point of the standard (7.8 pg/ml for MIF and 31.2 pg/ml for IL-8).

### Influence of MIF During *T. gondii* Infection in HTR8/SVneo Cells

In the third step of experiments, we investigated the effect of MIF on *T. gondii* intracellular proliferation by a colorimetric assay. For this purpose, HTR8/SVneo cells were cultured in 96-well plate (1 × 10^4^ cells/well/200 μl). After 24 h in culture, the cells were infected by *T. gondii* (3:1), and at the same time, they were treated with rMIF (2.5, 5, 15, 50, 100, and 200 ng/ml) or ISO-1 (200 μg/ml) for an additional 24 h. The cells were lysed with 100 μl of RIPA [50 mmol/L Tris hydrochloride, 150 mmol/L NaCl, 1% (v/v) Triton X-100, 1% (w/v) sodium deoxycholate, and 0.1% (w/v) SDS; pH 7.5] for 15 min, incubated with 160 μl assay buffer (100 mM PBS, 102 mM β-mercaptoethanol, and 9 mM of MgCl_2_) and 40 μl of CPRG (chlorophenol red-D-galactopyranoside; Roche Diagnostic, Manhein, Germany). The β-galactosidase activity was measured at 570 nm using a plate reader (Titertek Multiskan Plus, Flow Laboratories), and the data were shown as *T. gondii* intracellular proliferation index (number of tachyzoites) in comparison to a standard curve of free tachyzoites (1 × 10^6^ to 15.62 × 10^3^) (Challis et al., 2002; Castro et al., 2013; Barbosa et al., 2015). Five independent experiments were performed in six replicates.

### Western Blot

A Western blot was performed to detect CD74, CD44, ERK 1/2 phosphorylation, and COX-2 expression in total protein of HTR8/SVneo cells following different treatments. The cells were homogenized and lysed on ice in RIPA buffer; supplemented with a protease inhibitor cocktail (Complete®, Roche Diagnostic, Mannheim, Germany), 1 mM sodium orthovanadate (Na_3_VO_4_), and sodium fluoride (NaF) (both from Sigma); and subjected to three freeze-thaw cycles (Barbosa et al., 2014). After centrifugation at 10.621 × *g* for 30 min at 4°C, the supernatant was collected, and the concentration of total protein was measured by the Bradford assay (Bradford, 1976).

Total protein samples (150 μg) were subjected to polyacrylamide gel electrophoresis under denaturing conditions (SDS-PAGE), at 10%, and the proteins were electrotransferred to PVDF membranes (Millipore, Burlington, Massachusetts, USA). Blotted membranes were incubated in 4% fat dry milk in blotting buffer (17.52 g NaCl and 6.04 g Tris-buffered saline; pH 7.4) with 0.05% Tween for 1 h at room temperature and incubated overnight with the following primary antibodies: rabbit monoclonal anti-human CD74 (1:500, Santa Cruz Biotechnology®, Dallas, Texas, USA), rabbit monoclonal anti-human CD44 (1:200, Abcam®, Cambridge, UK), rabbit polyclonal anti-human phosphorylated ERK1/2 (1:1000, R&D Systems), goat polyclonal anti-human COX-2 (1:100, R&D Systems), or mouse monoclonal anti-β-actin (1:1000, Santa Cruz Biotechnology) in blotting buffer. Next, the membranes were exposed to the respective peroxidase-labeled secondary antibodies (1:3000 or 1:1000, Jackson Immuno Research Laboratories, West Grove, PA, USA) in blotting buffer with 2% fat dry milk for 2 h at room temperature. The reaction was revealed by chemiluminescence (ECL kit SuperSignal, Thermo Scientific, Waltham, Massachusetts, USA), and equal loading of the proteins was confirmed by staining the blots with 1% Ponceau. Densitometric analyses were performed for CD74, CD44, phosphorylated ERK 1/2 (phospho ERK1/2), and COX-2 to determine the mean intensity of the bands using the ChemiDoc (Bio-Rad, Berkeley, CA, USA). The data were presented as the relative density of the ratio between the proteins above described and β-actin. Nine independent experiments were performed in duplicate for each condition.

### Inhibition of ERK1/2 Activation

To confirm the role of ERK 1/2 in HTR8/SVneo cells, we performed experiments using PD98059, a potent ERK1/2 inhibitor. HTR8/SVneo cells were cultured in 96-well plates (1 × 10^4^ cells/well/200 μl). Next, the cells were infected with *T. gondii* (3:1) and treated (or not treated) with rMIF (200 ng/ml) and/or the ERK1/2 inhibitor (10 μM, PD98059) for 24 h. As a control, the infected cells were only treated with medium. Subsequently, β-galactosidase reaction assay was performed as described above.

### Statistical Analysis

Statistical analysis was performed using the GraphPad Prism 5.0 (GraphPad Software Inc., San Diego, CA, USA). All data were expressed as mean ± standard deviation (SD) or standard error of the mean (SEM). The comparisons of the data between groups were analyzed by one-way ANOVA, with the Bonferroni multiple comparison *post hoc* test or Student’s *t* test when appropriate. Statistical significance was established when the *p* < 0.05.

## Results

### MIF Is Upregulated by *T. gondii* Infection and Increased the Parasite Load in HTR8/SVneo Cells

**Figure 1:**
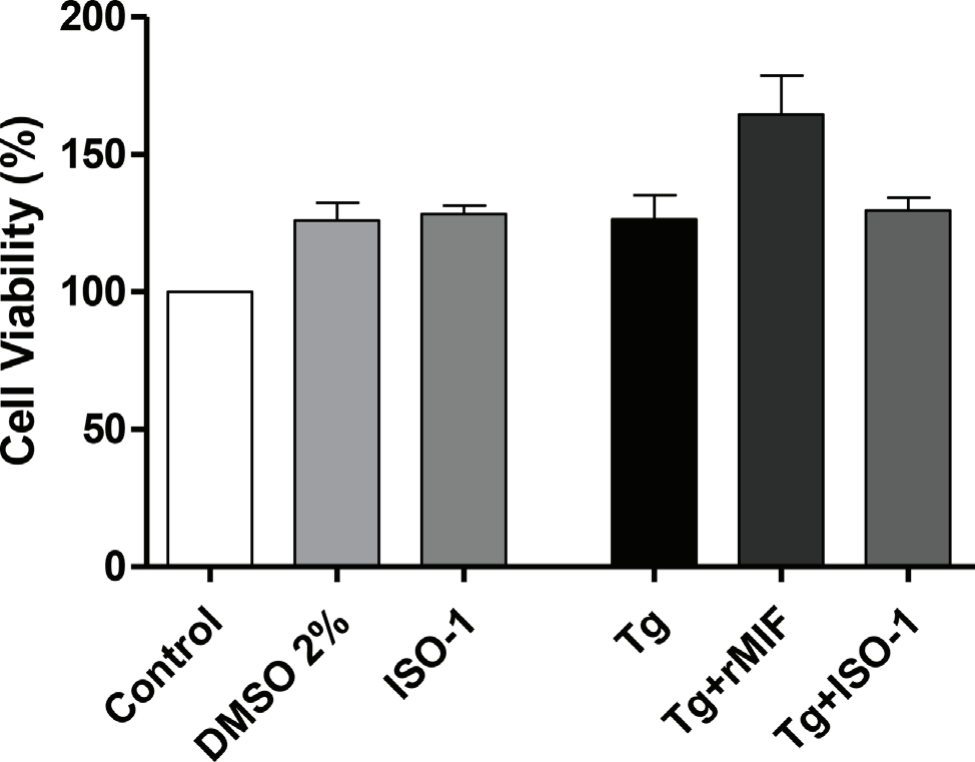
Viability of HTR8/SVneo cells. Cell viability was evaluated using a tetrazolium salt colorimetric (MTT) assay in *T. gondii*-infected (Tg) HTR8/SVneo cells treated with rMIF (Tg + rMIF) or ISO-1 (Tg + ISO-1) for 24 h. As a control, cells were only treated with medium (Control), with the vehicle (DMSO 2%) and with ISO-1 for 24 h. The data are expressed as the mean ± SEM of five independent experiments performed in triplicate. All data were analyzed by one-way ANOVA, with the Bonferroni multiple comparison *post hoc* test.

First, we verified the percentage of viable HTR8/SVneo cells after the various treatments and/or *T. gondii* infection. No significant differences were observed in cells treated with DMSO or ISO-1, in the absence of *T. gondii* infection when compared to uninfected/untreated cells only treated with medium (control). Also, infected/rMIF (Tg + rMIF) treated, infected/ISO-1 (Tg + ISO-1) treated, and infected/untreated (Tg) HTR8/SVneo cells did not present significant differences in cell viability when compared to control cells (Figure 1).

**Figure 2:**
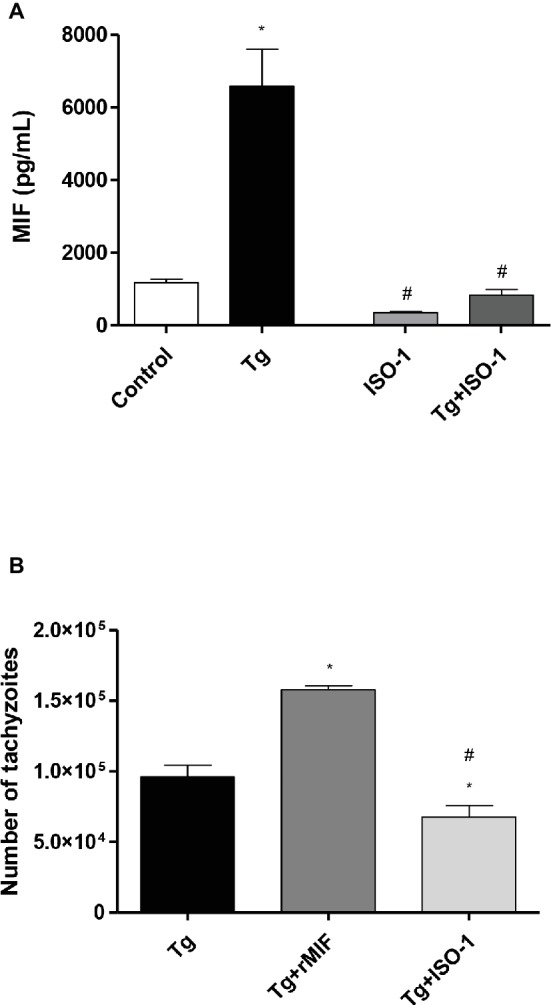
Extracellular MIF production by HTR8/SVneo cells and *T. gondii* intracellular replication. **(A)** Levels of extracellular MIF production by HTR8/SVneo cells infected with *T. gondii* (Tg) and/or treated with ISO-1 (Tg + ISO-1) or uninfected cells treated with ISO-1 alone (ISO-1). As a control, the cells were only cultured with medium (Control). The data are expressed as mean ± SEM and are representative of two independent experiments in triplicate. All data were analyzed by one-way ANOVA with the Bonferroni multiple comparison *post hoc* test. Significant differences in comparison with the Control (^*^p < 0.05) and Tg (^#^*p* < 0.05). **(B)** The *T. gondii* intracellular replication was analyzed by the β-galactosidase assay. HTR8/SVneo cells were infected with *T. gondii* and treated with rMIF (Tg + rMIF) or ISO-1 (Tg + ISO-1). As a control, HTR8/SVneo cells were infected and not treated (Tg). The data are expressed as the mean ± SEM of five independent experiments performed in triplicate. All data were analyzed by one-way ANOVA with the Bonferroni multiple comparison *post hoc* test. Significant differences in comparison with Tg (^*^p < 0.05) and Tg + rMIF (^#^*p* < 0.05).

Second, we investigated MIF production by ELISA, since this cytokine was shown to be important during *T. gondii* infection in the first trimester of pregnancy (Gomes et al., 2011). The data showed that Tg cells significantly increased the release of MIF when compared to control cells (*p* < 0.05, Figure 2A). Additionally, when the cells were treated with ISO-1, regardless of *T. gondii* infection, a decrease in MIF production was detected when compared to Tg cells (*p* < 0.05, Figure 2A). However, no significant difference was observed in MIF release between control cells and uninfected/ISO-1 (ISO-1) treated or Tg + ISO-1 treated cells (Figure 2A), or between ISO-1treated and Tg + ISO-1 treated cells (Figure 2A).

Third, intracellular proliferation of the parasite was analyzed in HTR8/SVneo cells with and without rMIF or ISO-1 treatments (Figure 2B). The Tg + rMIF treated cells showed an increase in the number of tachyzoites in comparison to Tg cells (*p* < 0.05, Figure 2B). We tested several rMIF concentrations (2.5, 5, 15, 50, 100, and 200 ng/ml) to determine if the effect was dose-dependent as previously demonstrated by Barbosa et al. (2014). The data showed, regardless of MIF concentration, that the number of tachyzoites was higher in Tg + rMIF treated cells than in Tg cells (data not shown). On the other hand, Tg + ISO-1 treatment significantly decreased the parasite number when compared with Tg or Tg + rMIF treated cells (*p* < 0.05, Figure 2B), showing the potential effect of ISO-1 in inhibiting MIF production.

### *T. gondii* and MIF Augment CD44 Expression in HTR8/SVneo Cells

**Figure 3:**
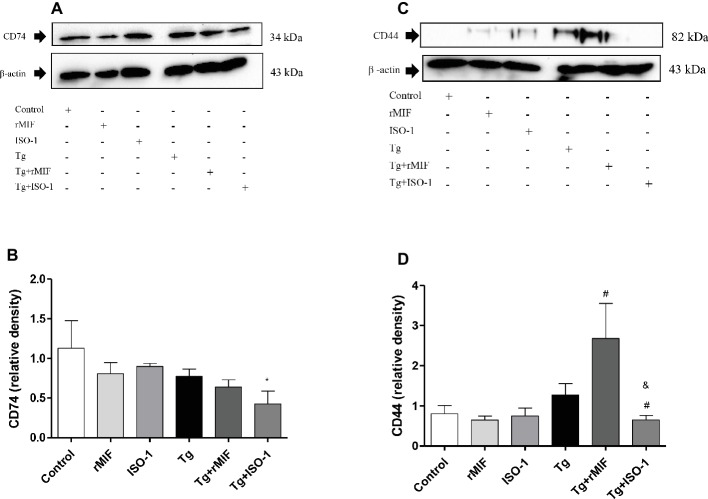
Detection of CD74 **(A–B)** and CD44 **(C–D)** in HTR8/SVneo cells. Cells treated with rMIF or ISO-1 for 24 h in the presence or absence of *T. gondii* infection were subjected to Western blot analysis to detect CD74, CD44, and beta-actin. As a control, cells were only treated with medium (Control) or infected and not treated (Tg). Representative Western blotting for CD74 **(A)**, CD44 **(C),** and their respective densitometric analysis **(B,D)** calculated from ratio between the proteins and beta-actin band. The data were expressed as the mean ± SEM of nine independent experiments performed in duplicate. All data were analyzed by one-way ANOVA with the Bonferroni multiple comparison *post hoc* test. **(A)** Significant differences in comparison with Control (^*^p < 0.05). **(B)** Significant differences between in comparison with Tg (^#^*p* < 0.05) and Tg + rMIF (^&^*p* < 0.05).

In order to understand the increase in *T. gondii* proliferation when HTR8/SVneo cells were treated with rMIF, we investigated the expression of the MIF receptor/co-receptor, CD74/CD44, since it has been shown that *T. gondii* can modulate and regulate the induction of CD74 expression in infected antigen presenting cells (Louis-Philippe et al., 2015) and the expression of CD44 in macrophages (Seipel et al., 2010; Hayashi et al., 2014). For this purpose, cells were cultured, treated, and infected by *T. gondii*, and the total proteins were used for immunodetection analysis of CD74 and CD44. CD74 expression remained unchanged in all experimental conditions, except in Tg + ISO-1 treated HTR8/SVneo cells, where lower levels of CD74 were detected when compared to control cells (*p* < 0.05, Figures 3A,B). However, treatment of infected cells with rMIF significantly increased CD44 expression in comparison to Tg cells (*p* < 0.05), while Tg + ISO-1 cells showed reduced CD44 expression when compared to Tg and Tg + rMIF treated cells (*p* < 0.05, Figures 3C,D).

### MIF Triggers Significant ERK 1/2 Phosphorylation in HTR8/SVneo Cells

**Figure 4:**
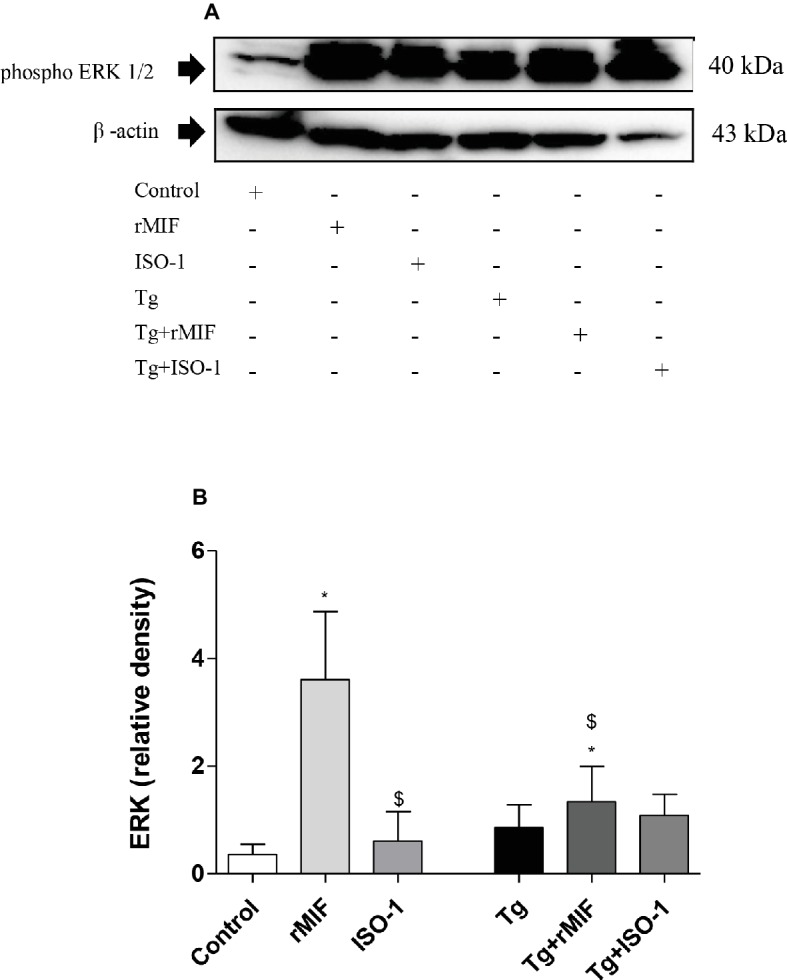
Detection of ERK1/2 phosphorylation in HTR8/SVneo cells **(A–B)**. Cells were treated with rMIF or ISO-1 for 24 h in the presence or absence of *T. gondii* infection and subjected to Western blot analysis to detect ERK 1/2 phosphorylation and beta-actin. As a control, cells were only treated with medium (Control) or infected and not treated (Tg). Representative Western blot for ERK 1/2 phosphorylation **(A)** and densitometric analysis **(B)** of the respective proteins calculated from the ratio between the phospho-proteins and beta-actin band. The data are expressed as the mean ± SEM of nine independent experiments performed in duplicate. All data were analyzed by one-way ANOVA with the Bonferroni multiple comparison *post hoc* test. Significant differences in comparison with Control (^*^p < 0.05). Significant differences with rMIF (^$^*p* < 0.05).

Next, we verified whether rMIF-triggered ERK 1/2 phosphorylation was involved in uninfected and *T. gondii*-infected HTR8/SVneo cells. Higher levels of ERK 1/2 phosphorylation were observed in uninfected/rMIF (rMIF) treated cells when compared to control cells (*p* < 0.05, Figures 4A,B). No significant difference was observed between ISO-1 treated and control cells (Figures 4A,B). By contrast, low levels of ERK 1/2 phosphorylation were observed in ISO-1 treated cells in relation to rMIF treated cells (*p* < 0.05, Figures 4A,B).

After infection, no significant differences were observed between the infected groups, regardless of treatment (Figures 4A,B). When we compared the infected group with uninfected group, higher levels of ERK 1/2 phosphorylation were observed in Tg + rMIF treated cells when compared to control cells, but a lower level of ERK 1/2 phosphorylation was detected in relation to rMIF treated cells (*p* < 0.05, Figures 4A,B).

### ERK 1/2 Phosphorylation Favors the *T. gondii* Infection in HTR8/SVneo Cells

Considering that activation of ERK 1/2 is MIF-dependent and favors parasitism in BeWo cells (Barbosa et al., 2014) and that the present data showed ERK 1/2 phosphorylation in HTR8/SVneo cells treated with rMIF, we evaluated whether ERK 1/2 phosphorylation is also required for *T. gondii* proliferation in human extravillous trophoblast.

**Figure 5:**
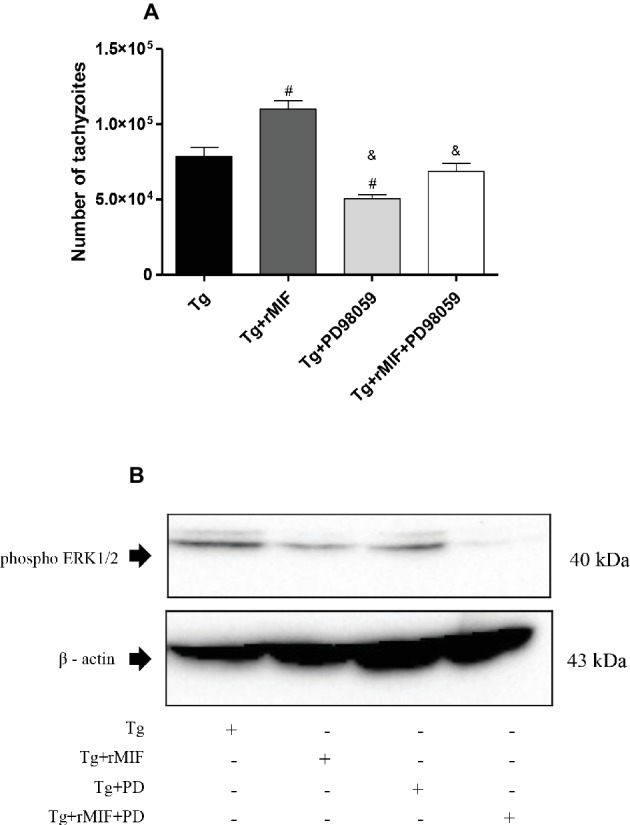
*T. gondii* proliferation in HTR8/SVneo cells and detection of ERK1/2 phosphorylation. **(A)** *T. gondii* intracellular replication was analyzed by the β-galactosidase assay. HTR8/SVneo cells were infected with *T. gondii* and treated with rMIF (Tg + rMIF), PD98059 (Tg + PD98059), or rMIF+PD98059 (Tg + rMIF + PD98059). As a control, HTR8/SVneo cells were infected and not treated (Tg). The data are expressed as the mean ± SEM of five independent experiments performed in triplicate. All data were analyzed by one-way ANOVA with the Bonferroni multiple comparison *post hoc* test. Significant differences in comparison with Tg (^#^*p* < 0.05) and Tg + rMIF (^&^*p* < 0.05). **(B)** Representative Western blot for ERK1/2 phosphorylation and beta-actin.

For this purpose, cells were infected with *T. gondii* and treated with ERK 1/2 inhibitor (PD98059) in the presence or absence of rMIF. We first performed the viability test for PD98059, and no significant difference in cell viability was observed in HTR8/SVneo cells treated with PD98059 (data not shown). As previously observed, a higher *T. gondii* intracellular proliferation in Tg + rMIF treated cells was observed when compared to Tg cells (*p* < 0.05, Figure 5A). On the other hand, the inhibitor of ERK 1/2 (Tg + PD98059) reduced *T. gondii* intracellular proliferation in comparison to Tg cells and Tg + rMIF treated cells (*p* < 0.05, Figure 5A). Also, infected/rMIF/PD98059 treated cells significantly controlled the number of tachyzoites in comparison to the respective control, Tg + rMIF (*p* < 0.05, Figure 5A). Furthermore, we confirmed that PD98059, at 10 μM, could inhibit ERK 1/2, since a significant reduction in ERK 1/2 phosphorylation in infected HTR8/SVneo cells, treated with PD98059 and/or rMIF, was detected in the presence of this inhibitor (*p* < 0.05, Figure 5B).

### COX-2 Expression Is Upregulated by *T. gondii* and MIF in HTR8/SVneo Cells

After verifying the association between rMIF, ERK1/2 phosphorylation, and the higher susceptibility to *T. gondii* in HTR8/SVneo cells, we finally investigated whether COX-2 was involved in this mechanism.

**Figure 6:**
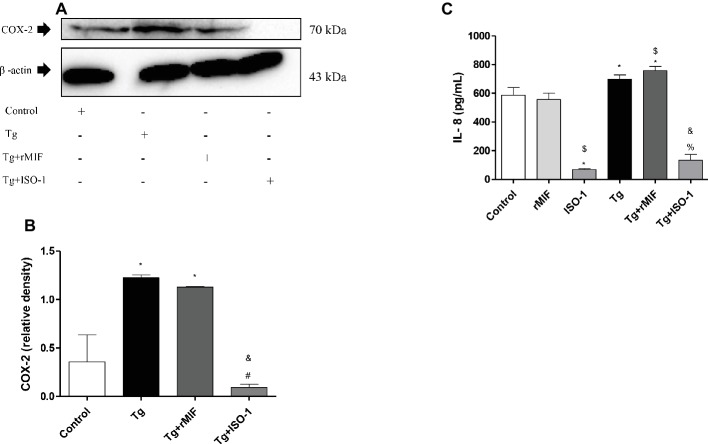
Detection of COX-2 **(A–B)** and IL-8 production **(C)** by uninfected or *T. gondii*-infected HTR8/SVneo cells. Untreated cells and cells treated with rMIF or ISO-1 in the presence or absence of *T. gondii* infection were subjected to Western blot analysis to detect COX-2 expression and beta-actin. Representative Western blot for COX-2 expression **(A)** and densitometric analysis **(B)** of the respective proteins calculated from the ratio between the COX-2 and beta-actin bands. The data are expressed as the mean ± SEM of nine independent experiments performed in duplicate. **(C)** In parallel, the cultured supernatants from cells treated with rMIF or ISO-1 for 24 h in the presence or absence of *T. gondii* infection were submitted to ELISA assay to analyze IL-8 production. As control, cells were treated with only medium (Control) or infected and not treated (Tg). The data were expressed as the mean ± SD of three independent experiments performed in duplicate. All data were analyzed by one-way ANOVA with the Bonferroni multiple comparison *post hoc* test **(B)** and Student’s test **(C)**. **(B)** Significant differences in comparison with the Control (^*^p < 0.05). Significant differences in comparison with Tg (^#^*p* < 0.05) and Tg + MIF (^&^*p* < 0.05). (**C**) Significant differences in comparison with Control (^*^p < 0.05). Significant differences in comparison with rMIF (^$^*p* < 0.05), ISO-1 (^%^*p* < 0.05), and Tg + rMIF (^&^*p* < 0.05).

Our data showed high COX-2 expression in Tg cells and Tg + rMIF treated cells when compared to control cells (*p* < 0.05, Figures 6A,B). Conversely, after ISO-1 treatment, infected cells showed a decrease in COX-2 expression in relation to Tg or Tg + rMIF treated cells (*p* < 0.05, Figures 6A,B). No significant difference was observed between Tg cells when compared Tg + rMIF treated cells (Figure 6B).

Next, we analyzed IL-8 production. Treatment with ISO-1 in uninfected cells reduced the secretion of IL-8 in comparison to control cells and rMIF treated cells (*p* < 0.05, Figure 6C). No significant difference was observed between control cells and rMIF treated cells (Figure 6C). The *T. gondii* infection increased IL-8 production in Tg cells when compared to control cells (*p* < 0.05, Figure 6C). Also, high IL-8 levels were observed in Tg + rMIF treated cells when compared to control and rMIF treated cells (*p* < 0.05, Figure 6C). The Tg + ISO-1 treated cells showed higher IL-8 production than ISO-1 treated cells (*p* < 0.05, Figure 6C). Conversely, Tg + rMIF treated cells exhibited higher levels of IL-8 than Tg + ISO-1 treated cells (*p* < 0.05, Figure 6C).

### Proposed Model of the Intracellular Mechanisms Triggered by MIF, ERK 1/2, COX-2, and IL-8 Production in HTR8/SVneo Cells During *T. gondii* Infection

**Figure 7:**
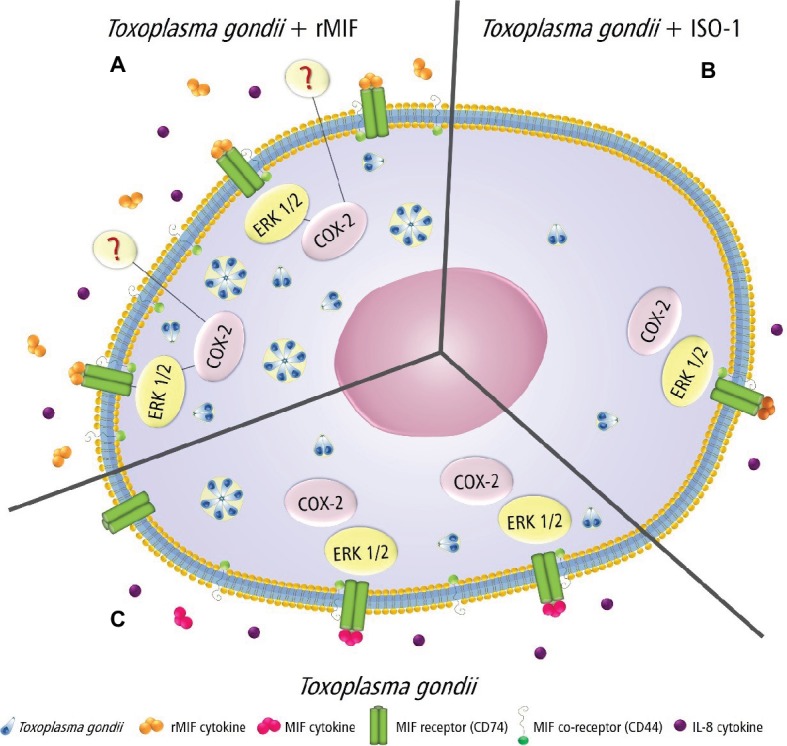
Proposed model showing the MIF effects in *T. gondii*-infected HTR8/SVneo cells. **(A)** rMIF produced by infected cells binds strongly to the CD74 receptor, phosphorylating the co-receptor (CD44), which in turn triggers the phosphorylation of ERK 1/2 and IL-8 production. The activation of ERK 1/2 activates COX-2, suggesting PGE_2_ production by HTR8/SVneo cells and increased parasitic proliferation. This mechanism could be used by *T. gondii* to proliferate and escape immune response in extravillous trophoblast cells. **(B)** HTR8/SVneo cells treated with ISO-1 decrease MIF release, and consequently, there is a reduction in its binding to a specific receptor (CD74), a decrease of CD44 expression, phosphorylation of ERK 1/2, COX-2 expression, and IL-8 production, leading to a lower parasite proliferation. **(C)** HTR8/SVneo cells infected with *T. gondii* secrete MIF, and this cytokine binds to its specific receptor (CD74) and phosphorylates its co-receptor (CD44), which in turn triggers the phosphorylation of ERK 1/2 and IL-8 production. The activation of ERK 1/2 activates COX-2 by HTR8/SVneo cells and increases *T. gondii* proliferation.

Based on our results, a proposed model of the effector mechanisms activated by MIF, ERK 1/2, COX-2, and IL-8 in HTR8/SVneo cells infected by *T. gondii* is shown in Figure 7. When HTR8/SVneo cells are infected and treated with rMIF, the upmodulation of the CD44 co-receptor, ERK 1/2 phosphorylation, the expression of COX-2, and the secretion of IL-8 were seen, suggesting the production of PGE_2_, and consequently, an increase in the number of *T. gondii* tachyzoites (Figure 7A). In the presence of ISO-1, MIF decreases, with subsequent reduced binding to the CD74 receptor, and decreased CD44 expression, phosphorylation of ERK 1/2, COX-2 expression, and IL-8 production, and consequently, a decrease in the number of *T. gondii* tachyzoites (Figure 7B). Finally, when HTR8/SVneo cells are infected with *T. gondii*, increased MIF production and phosphorylation of its co-receptor, CD44, trigger the phosphorylation of ERK 1/2 and induce IL-8 production. The activation of ERK 1/2 activates COX-2 in HTR8/SVneo cells and increases *T. gondii* proliferation.

## Discussion

Since its discovery in 1966 (Bloom and Bennett, 1966; David, 1966), MIF has proven to be an intriguing molecule of study for many scientists. This cytokine has a vital role in the reproductive immunology and inflammatory responses (Al-Abed et al., 2005). Also, MIF may play a critical role in activated macrophages killing some intracellular parasites, such as *T. gondii* (Jüttner et al., 1998). MIF is involved in several pathological conditions, such as infections (Das et al., 2013) and cancer (Du et al., 2013), explaining why therapeutic approaches aimed at inhibiting MIF activities use ISO-1 as a potential tool (Liang et al., 2010). In a previous study, our group demonstrated that MIF production by the human first-trimester placenta is upregulated by the *T. gondii* antigen, STAg, and may play an essential role, as an autocrine/paracrine mediator, in placental infection by this parasite (Ferro et al., 2008). In addition, we demonstrated previously that higher MIF production in human BeWo trophoblast cells and human villous explants, from the first- and third-trimester of pregnancy, infected by live tachyzoites of *T. gondii*, showed the potential control of this cytokine over the immune response to *T. gondii*, at the maternal-fetal interface (Ferro et al., 2008; Franco et al., 2011; Gomes et al., 2011). However, there are no studies showing the effect of MIF in human extravillous trophoblast cells. Thus, to verify the functional role of MIF in extravillous trophoblast, we investigated the expression of the MIF receptor/co-receptor, the parasite intracellular proliferation, and intracellular pathways triggered by MIF in HTR8/SVneo cells, an excellent *in vitro* model of extravillous trophoblast cells.

Initially, we checked the cytotoxicity of rMIF, ISO-1, and *T. gondii* in HTR8/SVneo cells. The choice of dosages for the cytotoxicity assay was performed based on previous studies using different concentrations of rMIF (10–200 ng/ml) and ISO-1 (1–200 μg/ml) in HTR8/SVneo cells, which were not shown to be toxic to the cells (Krivokúca et al., 2014). Therefore, the experimental concentrations for the cytotoxicity assay in this study were 200 ng/ml for rMIF and 200 μg/ml for ISO-1 (diluted in DMSO). Our data demonstrated that treatment with rMIF or ISO-1 was not cytotoxic to HTR8/SVneo cells, regardless of infection. These results agree with a study that used DMSO to dissolve drugs, in which diluting ISO-1 in DMSO did not decrease cell viability in HTR8/SVneo cells (Krivokúca et al., 2014). In another study, the AC16 cells (human cardiomyocytes) were treated with ISO-1 diluted in DMSO, and no decrease in cell viability was observed (Liang et al., 2010).

Once the concentrations were established, we investigated *T. gondii* infection in HTR8/SVneo cells treated with rMIF or ISO-1. Then, we investigated MIF extracellular secretion by HTR8/SVneo cells. Increased MIF production was detected in infected cells when compared to uninfected cells. By contrast, when HTR8/SVneo cells were treated with ISO-1, MIF production was significantly decreased, regardless of *T. gondii* infection. As expected, these results demonstrated the ability of ISO-1 to inhibit MIF production by HTR8/SVneo cells. Additionally, the present findings indicate the parasite’s ability to increase the production and release of the MIF cytokine. Some previous studies have demonstrated that *T. gondii* possesses many strategies of dissemination and survival in the host (Quan et al., 2015). One of these strategies is the induction of several types of cytokines in the host cells (Carvalho et al., 2010). An early study demonstrated the ability of *T. gondii* to induce higher levels of IL-12 and IL-23 in human monocytes (THP-1 cell line), which favored parasite survival in the host cells (Quan et al., 2015). Also, Maia et al. (2017) noted that secretion of IL-12 in patients with ocular toxoplasmosis may have been associated with the presence of *T. gondii* in the ocular region. In the maternal-fetal interface, we demonstrated that *T. gondii* can upregulate anti-inflammatory cytokines, such as IL-10 and TGF-β1, facilitating the infection of trophoblast cells (Franco et al., 2011). Finally, we also showed that MIF was upregulated in human trophoblast cells, and the control of *T. gondii* in these cells is MIF dose-dependent (Gomes et al., 2011; Barbosa et al., 2014).

After evaluating the higher MIF production by infected HTR8/SVneo cells, we verified parasitism in these cells under treatment with rMIF or ISO-1. Our results demonstrated that infected cells treated with rMIF showed a significant increase in parasite replication. Moreover, a significant reduction in parasite replication was observed when the infected cells were treated with ISO-1, proving the potent role MIF has in favoring *T. gondii* proliferation in HTR8/SVneo cells. However, the previous studies showed that MIF reduced the *Toxoplasma* infection in *in vivo* and *in vitro* experimental models. One group demonstrated that MIF promotes the classical activation of monocytes, promoting resistance to *T. gondii* infection in MIF^−/−^ mice treated with recombinant MIF (Ruiz-Rosado et al., 2016). Another study using *T. gondii*-infected MIF^−/−^ mice showed reduced lethality, as well as ileum inflammation and tissue damage, despite increased parasite intestinal load in relation to wild-type mice. The absence of MIF showed signs of systemic inflammation, including increased concentrations of plasma inflammatory cytokines and liver damage, suggesting that in susceptible hosts, MIF controls *T. gondii* infection at the cost of increasing local inflammation and systemic tissue damage (Cavalcanti et al., 2011). Furthermore, in the maternal-fetal interface, a study using chorionic villous explants suggested that MIF may play an important role in controlling *T. gondii* infection in the placental microenvironment (Ferro et al., 2008; Franco et al., 2011; Gomes et al., 2011). We previously demonstrated that MIF can control *T. gondii* proliferation in BeWo cells, when added at high concentrations. However, with low concentrations, the effect of MIF in reducing parasitism was insignificant (Barbosa et al., 2014). Conversely, we showed for the first time that low doses of MIF triggered increased *T. gondii* replication in human BeWo cells (Barbosa et al., 2014), and our present findings showed the same phenomenon in HTR8/SVneo cells. Thus, we can also conclude that MIF participates in mechanisms involved with *T. gondii* proliferation in HTR8/SVneo cells, increasing the susceptibility of this cell type.

The increased susceptibility of extravillous trophoblast cells to *T. gondii* is a mechanism still unknown; however, it can be associated to constitute the placenta. In humans and other mammals with hemochorial placentas, the maternal-fetal interface consists of the syncytiotrophoblast (SYN) that is bathed in maternal blood and mediates nutrient and gas exchange, and the extravillous trophoblasts (EVT) that anchor the placenta in the uterine implantation site (decidua) (Robbins et al., 2012; Ji et al., 2013). The SYN layer forms a protective barrier even at the earliest stages of pregnancy, with a complete layer of SYNs surrounding the embryo by the end of implantation. Once the utero placental circulatory system is fully established, which occurs near the end of the first trimester, the placenta is the sole barrier that prevents microorganisms from maternal blood to access the fetal compartment (Coyne and Lazear, 2016). Some studies have demonstrated that SYN layer is strongly resistant to infections and forms an effective barrier against the passage of some pathogens such as *T. gondii* and Zika virus, restricting the vertical transmission (Coyne and Lazear, 2016). However, the EVT cells may be an entry portal for microorganisms, enabling them to bypass the SYN barrier. In addition, these EVT cells are exposed to maternal blood during vascularization and the entire pregnancy course, in near contact with infected or uninfected maternal immune cells. By unknown mechanisms, it is not clear why EVT cells became more susceptible to *T. gondii* infection and more hospitable to parasite replication than the syncytiotrophoblast (Carlier et al., 2011; Robbins et al., 2012; Coyne and Lazear, 2016; Ander et al., 2018). In this sense, because the EVT cells are significantly more susceptible to *T. gondii* infection and parasite replication, the concentration of MIF used in the present study may favor parasite proliferation in HTR8/SVneo cells, since Barbosa et al. (2014) have already demonstrated it in human villous trophoblast cells (BeWo cell line). Therefore, it is plausible that MIF is beneficial to *T. gondii* in the extravillous trophoblast and that this cytokine has different action in modulating the parasite susceptibility according to the cell type. Future studies are needed to clarify the reason why extravillous trophoblast cells are more susceptible to pathogens when compared to other trophoblast populations or systemic cell types. Other intracellular signaling pathways, cytokines, chemokines, and enzymes should be investigated in order to compare why MIF can act in different manners in several trophoblast types or other mammalian cells. It is important to emphasize that trophoblast cells present different response to cytokines in comparison to other cell types, naturally. Our previous studies showed that human BeWo trophoblast cells are unable to control *T. gondii* replication when treated with exogenous levels of IFN-γ (Oliveira et al., 2006; Barbosa et al., 2008, 2015). IFN-γ is the most cytokine involved in the control of *T. gondii* infection in many mammalian cells (Gazzinelli et al., 1994), but it is not true for human trophoblast cells. Thus, it is notary that trophoblast cells are significantly different in relation to other mammalian cells.

In the present study, we demonstrated that an elevated MIF release occurred in the presence of the parasite. Thus, it is possible to speculate that MIF is secreted in response to infection, and it then associates with its receptor, signaling an intracellular pathway in response to infection. Considering this point, we examined the presence of the MIF receptor, CD74, in HTR8/SVneo cells and found that neither rMIF, ISO-1, nor *T. gondii* infection increased CD74 expression in these cells. These findings are in line with our previous study that demonstrated CD74 expression in human villous explant from the first trimester of pregnancy (Gomes et al., 2011) and BeWo cells (Barbosa et al., 2014). However, our data showed a decrease in CD74 expression in infected cells treated with ISO-1 when compared to the control, suggesting that this decrease in receptor expression could be a defense of the cell to control the parasitic proliferation. Certainly, many studies have demonstrated mechanisms used by cells to defend themselves against the proliferation of *T. gondii* such as stimulation of CD8^+^ T cells to secrete specific cytokines in the brain (Harris et al., 2012), the production of ROS (oxygen species) (Kim et al., 2017), and the elevated level of FasL in the supernatants of infected HTR8/SVneo cells and macrophages, preventing the spread of infection (Guirelli et al., 2015).

After evaluating CD74, we verified the presence of the co-receptor, CD44, in HTR8/SVneo cells, since some studies showed CD44 expression in the placenta (Bevilacqua et al., 2014) and their interaction with CD74 (Subbannayya et al., 2016). Our data demonstrated that rMIF treatment, together with *T. gondii* infection, increased CD44 expression. Studies demonstrated that CD44 expression was decreased on the surface of *T. gondii*-infected macrophages, modulating their migration (Seipel et al., 2010). Alternatively, in leukocytes (Hayashi et al., 2014) and monocytes, CD44 expression was increased as a mechanism used by the parasite to reach the bloodstream and reach the peripheral organs (Unno et al., 2010). Thus, it can be assumed that *T. gondii* can modulate CD44 expression in the cells to disseminate in the host. Due to the knowledge that the strong binding between MIF and CD44 triggers an intracellular signaling pathway, we wanted to examine the presence of ERK 1/2 phosphorylation in HTR8/SVneo cells and its relation to the increase of *T. gondii* proliferation in these cells. In the present study, the data demonstrated that rMIF treatment, regardless of infection, increased ERK 1/2 phosphorylation. Other studies have shown that MIF exerts its functions through activation of several signaling pathways, including ERK 1/2 (Subbannayya et al., 2016). Our previous study showed that MIF participates in the immune response to *T. gondii* and triggers ERK 1/2 phosphorylation and PGE_2_ release in BeWo cells (Barbosa et al., 2014). Also, another study demonstrated that the activation of ERK 1/2 is induced for protein kinase A and associated with cytoplasmic phospholipase A2 (PLA_2_) enzyme activity (Calandra et al., 1995). PLA_2_ is an important intracellular protein that induces production of arachidonic acid and then PGE_2_ and leukotrienes (Calandra et al., 1995).

Since *T. gondii* has the ability to utilize the MIF cytokine together with its co-receptor to trigger the intracellular pathway of ERK 1/2 and consequently increase parasitic proliferation, we assessed whether inhibiting the ERK 1/2 pathway would reduce *T. gondii* proliferation. We observed reduced *T. gondii* proliferation in HTR8/SVneo cells treated with the ERK 1/2 inhibitor, PD98059. The use of this specific inhibitor for MAP kinase also suggests that ERK 1/2 and PGE_2_ participate in the mechanisms of parasite replication in HTR8/SVneo cells. These findings agree with our previous study, demonstrating that the intracellular mechanism triggered by MIF is dose-dependent in BeWo cells, and PGE_2_ is a key factor for the persistence of *T. gondii* at the maternal-fetal interface (Barbosa et al., 2014).

Next, we verified the COX-2 expression in HTR8/SVneo cells, since this signaling pathway is related to ERK 1/2 and the production of PGE_2_ (Kniss et al., 2001). Our results demonstrated that HTR8/SVneo cells, when infected or infected and treated with rMIF, there was an increase in COX-2 expression. Thus, it is possible to speculate that increased parasite proliferation could be related to increased expression of the co-receptor CD44, which consequently phosphorylates ERK 1/2, and this stimulates COX-2 in infected and rMIF-treated HTR8/SVneo cells.

Some studies established the relationship between MIF, CD74, CD44, MAPK (ERK 1/2), and overexpression of PGE_2_, essential for reproduction, inflammation, and endometrium reconstruction (Mahdian et al., 2015). The expression of COX-2 and PGE_2_ is also associated with the activation of signaling pathways, such as p38, JNK, and/or ERK 1/2 MAPKs during the invasion and proliferation of *T. gondii* tachyzoites, increasing secretion and expression of the monocyte chemoattractant protein (MCP-1) and macrophage inflammatory protein 1-alpha (MIP-1α) in infected macrophages (Kim et al., 2006). Thus, we can conclude that COX-2 expression in HTR8/SVneo cells is dependent on MIF/CD44 and ERK1/2 phosphorylation.

Lastly, we evaluated the effect of IL-8 produced by HTR8/SVneo cells infected, in the presence or absence of rMIF and ISO-1 treatments. It was previously reported that IL-8 was present in serum and increased in patients with *T. gondii* infection (Rostami Nejad et al., 2011). It was also demonstrated that *T. gondii* only induces a significant increase in IL-8 secretion when compared with *Trypanosoma cruzi* infection in human placental chorionic villi explants (Castillo et al., 2017). In the present study, we showed that IL-8 secretion was increased in infected HTR8/SVneo cells treated with rMIF. These findings coincide with some studies that revealed that MIF markedly upregulates vascular endothelial growth factor (VEGF), IL-8, and MCP-1 expression in endometriotic cells *via* CD74 and MAPK signaling pathways (Mahdian et al., 2015). Also, IL-8 mRNA upregulation was reported in *T. gondii*-infected HeLa cells and human fibroblasts (Taubert et al., 2006), and *T. gondii* synthesized a MIF homolog, called TgMIF, which was able to induce IL-8 production in human peripheral blood mononuclear cells (PBMCs) (Sommerville et al., 2013). These MIF homologues appear to be involved universally in innate and adaptive immune responses and affect cell migration, pro-inflammatory cytokine secretion, and cell differentiation or morphogenesis (Bozza et al., 2012). TgMIF protein from *T. gondii* can activate the ERK 1/2 pathway in mononuclear cells, and TgMIF is used to modulate the host immune response, thereby favoring parasite invasion/maintenance (Sommerville et al., 2013). Thus, it is possible to speculate that *T. gondii* uses MIF to manipulate the ERK 1/2 signaling pathway and IL-8 to increase its proliferation in HTR8/SVneo cells.

In conclusion, the present study demonstrated that *T. gondii* increased MIF production by HTR8/SVneo cells, and it caused CD44 upregulation that activated the ERK 1/2-intracellular signaling pathway, leading to increased COX-2 expression and IL-8 production. In this regard, *T. gondii* can modulate key factors in HTR8/SVneo cells and this can explain the higher susceptibility of extravillous trophoblast cells to *T. gondii* infection when compared to other trophoblast cell populations.

## Data Availability

The raw data supporting the conclusions of this manuscript will be made available by the authors, without undue reservation, to any qualified researcher.

## Author Contributions

IM and EF designed the experiments. RS, CM-M, BB, PG, MR, AG, FI, PF, and IM performed the experiments. RS, BB, AG, PF, and IM analyzed the data. BB, JM, and EF provided the reagents. IM, RS, PG, PF, and EF discussed the findings. BB, PF, JM, and EF reviewed the manuscript. All authors approved the final version of the manuscript.

### Conflict of Interest Statement

The authors declare that the present work was carried out in the absence of any personal, professional or financial relationships that could potentially be construed as a conflict of interest.
